# Chemistry towards Biology—Instruct: Snapshot †

**DOI:** 10.3390/ijms232314815

**Published:** 2022-11-26

**Authors:** Miloš Hricovíni, Raymond J. Owens, Andrzej Bak, Violetta Kozik, Witold Musiał, Roberta Pierattelli, Magdaléna Májeková, Yoel Rodríguez, Robert Musioł, Aneta Slodek, Pavel Štarha, Karina Piętak, Dagmara Słota, Wioletta Florkiewicz, Agnieszka Sobczak-Kupiec, Josef Jampílek

**Affiliations:** 1 Institute of Chemistry, Slovak Academy of Sciences, Dúbravská cesta 9, 845 38 Bratislava, Slovakia; 2 Structural Biology, The Rosalind Franklin Institute, Harwell Science Campus, UK, University of Oxford, Oxford OX11 0QS, UK; 3 Division of Structural Biology, The Wellcome Centre for Human Genetics, University of Oxford, Oxford OX3 7BN, UK; 4 Institute of Chemistry, University of Silesia, Szkolna 9, 40 007 Katowice, Poland; 5 Department of Physical Chemistry and Biophysics, Pharmaceutical Faculty, Wroclaw Medical University, Borowska 211A, 50 556 Wrocław, Poland; 6 Magnetic Resonance Center and Department of Chemistry “Ugo Schiff”, University of Florence, 50019 Sesto Fiorentino, Italy; 7 Center of Experimental Medicine SAS and Department of Biochemical Pharmacology, Institute of Experimental Pharmacology and Toxicology, Slovak Academy of Sciences, Dubravska cesta 9, 841 04 Bratislava, Slovakia; 8 Department of Natural Sciences, Eugenio María de Hostos Community College, City University of New York, 500 Grand Concourse, Bronx, NY 10451, USA; 9 Department of Pharmacological Sciences, Icahn School of Medicine at Mount Sinai, 1425 Madison Avenue, New York, NY 10029, USA; 10 Department of Inorganic Chemistry, Faculty of Science, Palacký University Olomouc, 17. listopadu 1192/12, 771 46 Olomouc, Czech Republic; 11 Department of Materials Science, Faculty of Materials Engineering and Physics, Cracow University of Technology, 37 Jana Pawła II Av., 31 864 Krakow, Poland; 12 Department of Analytical Chemistry, Faculty of Natural Sciences, Comenius University, Ilkovičova 6, 842 15 Bratislava, Slovakia

**Keywords:** chemical biology, biological chemistry, molecular interactions, structure and dynamics, targeting, virtual screening, proteins and nucleic acids, natural compounds, biomolecules, biomaterials

## Abstract

The knowledge of interactions between different molecules is undoubtedly the driving force of all contemporary biomedical and biological sciences. Chemical biology/biological chemistry has become an important multidisciplinary bridge connecting the perspectives of chemistry and biology to the study of small molecules/peptidomimetics and their interactions in biological systems. Advances in structural biology research, in particular linking atomic structure to molecular properties and cellular context, are essential for the sophisticated design of new medicines that exhibit a high degree of druggability and very importantly, druglikeness. The authors of this contribution are outstanding scientists in the field who provided a brief overview of their work, which is arranged from in silico investigation through the characterization of interactions of compounds with biomolecules to bioactive materials.

## 1. Introduction

The knowledge of interactions between different molecules is undoubtedly the driving force of all contemporary biomedical and biological sciences. Chemical biology/biological chemistry has become an important multidisciplinary bridge connecting the perspectives of chemistry and biology to the study of small molecules/peptidomimetics and their interactions in biological systems [1,2]. The success of all multidisciplinary fields and approaches is based on communication. For this reason, the conference series called Central European Conference “Chemistry towards Biology” was born, and exactly 20 years ago, in 2002, the first meeting took place in Portoroz, Slovenia. Since then, the conference has been held every two years in one of the cities of Central Europe [3]. In September 2020, the conference had to be canceled due to the COVID-19 pandemic but returned this year to celebrate its tenth anniversary. We are very pleased that the Chemistry towards Biology conferences have survived the difficult period of the pandemic and that the tradition of the conferences has been maintained. The aim of the series is to promote the exchange of scientific results, methods, and ideas and encourage cooperation between researchers from all over the world. The topics of the conferences cover Chemistry towards Biology, meaning that the events welcome chemists working on biology-related problems, biologists using chemical methods, and students and other researchers of the respective areas that fall within the common scope of chemistry and biology.

The tenth year of the “Chemistry towards Biology” conference (CTB10) was held in Bratislava, Slovak Republic, on 11–14 September 2022 at the same time as the European Infrastructure Instruct Meeting. The Instruct Consortium [4] supports advances in structural biology research, especially the connection between atomic structure and molecular properties in a cellular context. The topics of “Chemistry towards Biology 10—Instruct” meeting [5] were the structure and dynamics of biomolecules, intermolecular interactions, and experimental and theoretical methods in biomolecular research. In total, 93 active participants from 10 countries around the world presented their novel results. The authors of this manuscript are plenary speakers, other important participants of the symposium, and members of their research teams. The following summary highlights the major points and topics of the symposium. Individual reports/sections are arranged from in silico investigation through the characterization of interactions of compounds with biomolecules to bioactive materials. Section 2 and Section 3 cover general aspects of drug design (cheminformatics and physicochemical profiling of potential drugs). The next three parts (4–6) discuss the analysis of proteins that have been found to be important for target-oriented drug design, i.e., targeted therapy (antiviral antibodies, anticancer drugs), or drug design against major diseases (cardiovascular, neurodegenerative, inflammatory). Section 7, Section 8 and Section 9 deal with the preparation of bioactive compounds based on metal complexes with either anticancer potential or as fluorescent probes for use in diagnostics. The final section describes promising biocompatible polymer-ceramic composites applicable as drug carrier systems or implants.

In 2024, the 11th Central European Conference “Chemistry from Biology” will be held in Krakow (Poland) under the patronage of Professor Grazyna Stochel from the Faculty of Chemistry of the Jagiellonian University, to which everyone is cordially invited.

## 2. Similarity-Mediated Property Profiling in Drug Design

The most fundamental aim of medicinal chemistry is to rationalize decision making in the pathway of hit identification→lead optimization→drug nomination [6]. Finding a ‘sweet spot’ (the critical in-vivo/vitro/silico descriptors or properties) on the route towards the ‘prediction paradise’ requires at least four German G’s: Glück (luck), Geld (money), Geschick (skill) and Geduld (patience) [7]. Fortunately, the medicinal chemist’s intuition (or serendipity) at the pre-synthetic stage can be supported by computer-aided molecular design (CAMD) reducing the risk of drugs failing late in the development process. In other words, the concept of ‘fail-early fail cheaply’ is employed [8]. A range of in silico methods have been introduced for mapping the molecular topology/topography that are encoded with the symbolic/numeric descriptors into the property-based chemical space (CS). However, the straightforward transition from intricate biological relations into simple quantitative structure-activity relationships (QSARs) is rather a ‘triumph of hope over experience’ [9]. On the other hand, SAR-guided mining of descriptor-based space has become a typical procedure on the route from data to drugs with ADMET-tailored properties, especially for congeneric series of molecules.

Molecular similarity is at the core of many SAR-related methods, but the validity of such methods is questionable since there is no standard measure of similarity. Moreover, similarity is a subjective concept related to different aspects of human cognition—a phenomenon called ‘psychological proximity’, because similarity depends on the ‘eye of the beholder’ [10]. Nonetheless, the idea of specifying a numerical measure of inter-molecular similarity is still widely applied in SAR studies. Systematic observation of structural modifications and the corresponding response variations (e.g., biological activity) for similar compounds is a ‘gold standard’ in computational chemistry. However, the practical development of a global model for a diverse set of molecules is problematic.

**Figure 1:**
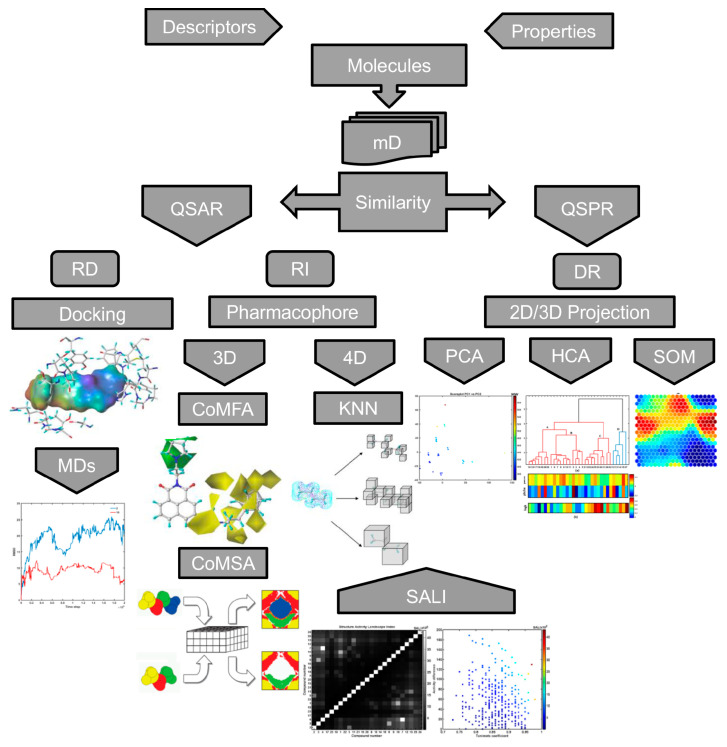
Workflow of descriptor-based data in similarity-related property modeling (details in text).

Generally, computer-assisted manipulation of drug-receptor interactions can be divided into ‘indirect’ (ligand-based) and ‘direct’ (structure-based) procedures as shown in Figure 1. The qualitative and/or quantitative rationalization of the drug–target binding forces in the receptor-dependent (RD) procedures can be partially achieved using site-directed molecular docking and dynamic simulations (MDs). However, the binding system evaluation is still questionable due to a deficiency in truly selective scoring functions [11,12]. Theoretically, the receptor-independent (RI) approach stems loosely from the similarity principle, where interchangeable steric/electronic/lipophilic-like substituents are bound to exert a similar impact on the pharmacological profile (neighbor behaviors) [13]. In practice, the ‘reverse image’ of the hypothetical target binding geometry is generated for the ensemble of structurally related (bio)molecules in the form of a pharmacophoric pattern. This in turn can specify a spatial (3D) distribution of molecular features that are necessary, but not sufficient for biological activity [14]. In fact, a wide range of 3D-QSAR procedures have been practically implemented in the field of medicinal/computational chemistry using molecular interaction/energy field (e.g., CoMFA), molecular surface/volume (e.g., CoMSA) descriptors, respectively [15]. Comparative molecular field analysis (CoMFA) is historically the first method that allows modeling of the influence of molecular shape on steric (Lennard–Jones) and electrostatics (Coulomb) on non-covalent ligand–receptor interactions. Roughly speaking, CoMFA assumes that variations in binding affinities for structurally related compounds can be explained by a comparison of 3D field-based patterns produced within the cubic mesh of points, which encompasses aligned molecules using the selected probe atoms [16]. A number of alternative CoMFA-like protocols have appeared, e.g., comparative molecular surface analysis (CoMSA) that implemented corrections in the molecular shape description, superimposition rules as well as predictive model quality [17]. CoMSA replaces potential values calculated at single points with the mean potential values specified for surface sectors; therefore the ‘fuzzification’ of the molecular shape representation is achieved. In practice, the rough quantitative comparison of the field-based and surface-related descriptors can provide a more realistic picture of the ligand–target recognition scenario, though the question about the underlying biological reality remains unanswered.

Obviously, a molecule is a dynamic object and therefore the alignment problem is the ‘Achilles heel’ of field/surface-based protocols, especially for conformationally flexible systems. Practically, the postulated ‘bioactive’ 3D ligand conformation is constructed as a ‘sophisticated guess’ (not necessarily geometry-optimized ones). Hence, 4D-QSAR schemes have been implemented to give a higher level of a model abstraction using procedures that allow the construction of optimized dynamic spatial QSAR models. These are in the form of 3D pharmacophores, which are dependent on conformation, alignment, and pharmacophore-grouping’ [18]. In fact, 4D-QSAR can be regarded as a variant of molecular similarity estimation in the molecular shape analysis (MSA), where the substitution of the ‘explicit’ atom-based compound pattern with the ‘implicit’ cube-alike population generates ‘fuzzy’ molecular representation [19]. The conventional cell-based Hopfinger’s 4D-QSAR coding system employs an ensemble of cubic shape-like descriptors that are calculated for the multiple molecule conformational/alignment states as the ‘fourth pseudo-dimension’ [20]. The extension of the classical grid cell occupancy descriptors (GCODs) with charge ones was proposed with absolute, joint, and self-charged descriptors, respectively. Moreover, a neural formalism employing self-organizing maps (SOMs) to generate ‘fuzzy’ 4D-QSAR-like representations of conformational space has been proposed, namely SOM-4D-QSAR [21,22]. The adaptive and competitive Kohonen SOM (KNN) algorithm is used to generate planar (2D) topographic maps, that represent the signals from chosen atoms of the molecular trajectory.

In QSAR studies, a ‘fragile event’ might occur, when even a tiny structural modification (termed ‘magic methyl’) can boost or completely demolish the biological activity—a phenomenon known as the activity ‘hotspot’ or ‘activity cliff’ in the structure–activity landscape [23]. The optimal balance between ADMET-tailored properties and the expected drug potency profile can be rationalized graphically by enhancement of the planar similarity-driven projection with activity data in the form of the ‘response surface’ or SAR landscape. Detection of similarity-based SAR trends (smooth or flat regions and sharp or non-uniform areas) using 2D images of the structure–activity landscape indexes (SALI) depends critically on the availability of structurally similar molecules (chemotypes) with discernible variations in activity [24]. The systematic profiling of a potency-similarity landscape provides a subtle picture of (un)favorable structural modifications that can help to modulate pharmacological response and optimize ADMET-friendly drug properties.

Unfortunately, most of the topological/topographical descriptors are highly inter-correlated. Therefore, linear (e.g., principal component analysis, hierarchical clustering analysis) or/and non-linear (e.g., self-organizing maps) data reduction (DR) procedures need to be employed in order to illustrate the molecular similarity in the 2D/3D space. The distance-oriented property evaluation can be conducted using principal component analysis (PCA), where the original descriptor-based data are decomposed and molecules (usually color-coded by the selected properties) are projected onto planes defined by the explanatory (orthogonal) principal components (PCs). Further, exploratory hierarchical clustering analysis (HCA) can be performed to investigate the (dis)similarities between objects (molecules) in the multidimensional descriptor-based space [25]. The clustering tendency of HCA leads to a sub-optimal grouping of objects, that is mostly related to the procedure engaged for cluster linkage. Due to the hierarchical nature of the HCA method, the results are presented as dendrograms generated in Euclidean-based distance, where the *x* axis presents the sequence of objects/parameters, and the *y* axis specifies the dissimilarity. Usually, the interpretability of the extracted data structure is not simple in the multidimensional variable space; therefore, the dendrogram might be augmented with a color-coded map of the experimental data (see Figure 1). Finally, self-organizing Kohonen neural mapping (SOM) is a nonlinear projection procedure that reduces the input data dimensionality (e.g., converts 3D objects to 2D), while preserving the topological relationships between the input and output data. Moreover, a trained network can be engaged to project the specified molecular property (expressed as a vector) by generating a 2D color-coded clustering pattern called a feature map.

In conclusion, the quantitative atom-based (CoMFA) and shape-related (CoMSA) ligand-oriented sampling of inter-molecular similarity with the generation of a pharmacophore pattern, is valid to illustrate the key 3D steric, electronic, and lipophilic features of the ligand–receptor composition. The detection of activity ‘hotspots’ provides valuable hints on how to produce potentially more potent drug candidates. Therefore, the numerical quantification of activity cliffs is frequently performed in the SAR-driven similarity evaluation of molecular properties using a variety of fingerprint representations and/or similarity metrics. Moreover, the descriptor-based similarity assessment of property space can be performed using PCA, HCA, and SOM methods, respectively.

## 3. Potential of Langmuir Balance and Isotherms in Research and Development of New Pharmaceuticals

Research performed with the Langmuir balance is based mainly on surface tension measurements and presentation of specific Langmuir isotherms. The surface pressure is plotted as a function of the surface on which the monomolecular layer of surfactant is dispersed. Evaluation of the isotherms leads to important conclusions, including the approximated size of assessed particles, and the relationships between the structure and spatial arrangement of the particles in the monolayer. The pioneering works of Ludwig Wilhelmy, Irving Langmuir, and Katharine Burr Blodgett paved the way for current methods of evaluating the behavior of monolayers under various conditions. The development of modern methods for the evaluation of isotherms has enabled the application of Langmuir balance to studies of new drugs or medicinal products.

A literature survey was carried out to identify the most important and interesting applications of the Langmuir balance in research on new medicinal substances or medicinal products. Publications in such fields of applied science as: drug analysis, drug chemistry, pharmaceutical chemistry, drug synthesis, drug technology, pharmaceutical formulation, pharmacokinetics, biopharmacy and pharmacology, and drug delivery were analyzed. Papers dealing with these topics were extracted from the world wide web via a commercially available web browser with a function for recognition of scientific publications. The perspective of ten years was applied, i.e., the years 2012–2021.

The highest number of applications of Langmuir balance was revealed to be drug chemistry, closely followed by drug analysis, drug technology, mainly development of synthetic processes and drug synthesis. Some specific aspects of pharmaceutical science, e.g., pharmaceutical formulation, drug delivery, or pharmacokinetics, were sparsely represented, with the lowest number of publications in the field of biopharmacy, which is partially synonymous with pharmacokinetics. Detailed information on the fraction of selected applications of Langmuir balance is given in Table 1.

**Figure 2:**
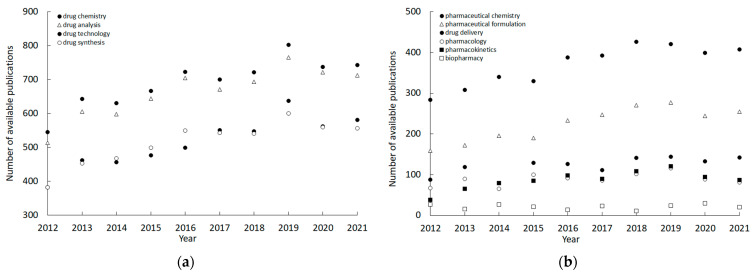
The number of available publications in the fields which add to over 50% coverage of the application of Langmuir balance (**a**), and below 50% coverage of the application of Langmuir balance (**b**).

The number of papers in the evaluated field increases in most cases year by year, as it is known from other disciplines; however, the increase in the fields recognized as more connected to the practical aspects of drug production or application is less pronounced, as shown in Figure 2.

Among the applications of Langmuir balance in drug development, were studies in which the interaction or influence of a drug substance on the properties of the monolayer was assessed, e.g., curcumin [26] and penicillamine [27]. Another category of use was the analysis of the interactions of nano-scale polymer particles—potential drug carriers—with lipid layers [28]. The influence of radiation, pH, and/or electrolytes on the properties of a monolayer obtained from a specific substance with surface-active properties, e.g., with the use of dipalmitoylphosphatidylcholine [29], has been investigated. An interesting area of research has been attempts to use the monolayer as a model of the cell membrane, e.g., in the study of violacin [30], or as a model of tissue fluid, e.g., tear fluid [31]. Finally, a large number of papers dealt with the natural surfactants present in the human pulmonary system [32].

In conclusion, studies using the Langmuir balance remain an important option for the modeling and development of substances and medicinal products with a component that have a pronounced effect on the surface tension of a solution.

## 4. Structural and Functional Analysis of Nanobodies to the Spike Protein of SARS-CoV-2

There are currently seven known coronaviruses that infect humans of which three (SARS-CoV-1, MERS-CoV, SARS-CoV-2) have emerged in the last 20 years and caused severe and even fatal respiratory diseases. By far the most serious outbreak has been caused by SARS-CoV-2 which has been responsible for 6.5 million deaths worldwide, with massive economic dislocation and long-term health consequences. Although vaccines are now available for SARS-CoV-2, building up immunity in the global population will still take years. This would be further delayed by variants of concern, which may cause vaccine breakthrough. Therefore, significant effort has been invested by many groups to produce effective anti-viral treatments including the use of antibodies for passive immunotherapy. As an alternative to conventional antibodies, camelid-derived nanobodies (VHHs) offer advantages in terms of stability and production costs in microbial systems whilst retaining high affinity and specificity.

A nanobody to the receptor binding domain (RBD) of SARS-CoV-2 (Wuhan) was isolated by screening a naïve library of llama VHHs. Binding affinity was increased from micromolar to low nanomolar by random mutagenesis of the third complementary determining region (CDR3). The affinity-matured nanobody blocked the binding of isolated RBD to angiotensin-converting enzyme-2 (ACE-2), the cell surface receptor required for virus entry, and neutralized live viruses in a cell-based infection assay [33]. The underlying basis for affinity improvement was investigated by solving the structures of several nanobodies derived from the same parental sequence in complex with either the isolated RBD or spike protein using X-ray crystallography and cryo-electron microscopy (cryo-EM), respectively [34]. Isothermal calorimetry confirmed that the interaction between the nanobodies and both the spike and RBD was enthalpically driven and entropically unfavorable. A computational analysis of the ensembles of structures generated by cryo-EM using the electron meta-inference method [35,36] showed a reduction in the conformational dynamics of the nanobody RBD complexes with increasing affinity. This insight was used to design a mutant nanobody that had improved binding to the spike protein due to reduced entropic penalty [34].

**Figure 3:**
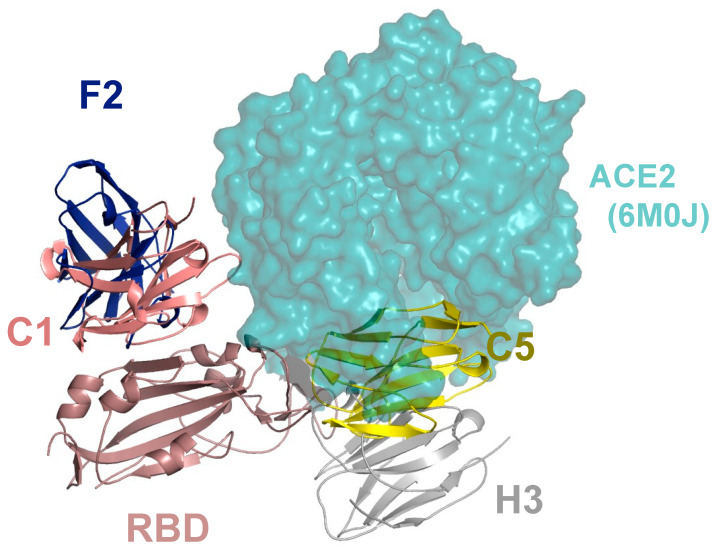
Montage of RBD–nanobody X-ray complexes with location of ACE-2 binding. The four nanobodies are shown in cartoon and labelled. The figure was generated by superimposing the RBD protein from each crystal structure, only one RBD monomer is shown. Also shown is ACE2 (cyan surface) from the RBD ACE2 complex (PDB 6M0J), positioned by superposition of the RBD.

A second-generation series of nanobodies was produced by immunization of a llama with a combination of RBD and spike proteins. Four nanobodies were selected and showed significantly higher affinity and virus neutralization activity compared to the nanobody identified from a non-immunized library. The binding epitopes of these nanobodies were mapped by determining the structures of nanobody–RBD complexes and were shown to localize to either the side of the RBD, distal from the ACE-2 receptor binding interface (nanobodies C1 and F2) or close to the ACE-2 binding region (nanobodies H3 and C5) (Figure 3).

This information was used to design a sensitive sandwich ELISA for detecting both isolated spike protein and inactivated SARS-CoV-2 viruses [37]. The structural data also enabled the rationalization of the results from neutralization studies with different variants of concern. Further, the therapeutic potential of anti-RBD nanobodies was shown by treatment with a single dose of the most potent nanobody (C5), either systemically (intraperitoneal route) or via the respiratory tract (intranasal route) which led to the prevention of disease progression in the Syrian hamster model of COVID-19 [38].

Coronaviruses seem especially prone to jump the species barrier and the emergence of future highly impactful coronaviruses in humans seems possible. A new coronavirus that was not neutralized by antibodies generated by COVID-19 infection or vaccine, would pose a significant pandemic risk. Therefore, assembling nanobody reagents that have broad cross-reactivity against different lineages of Beta-coronaviruses is the focus of ongoing work.

## 5. Just Flexible Linkers?

Intrinsically disordered regions (IDRs) of multi-domain proteins have been for a long time considered just simple linkers connecting functional globular domains and thus ignored in structural biology studies. However, in many cases, they comprise a significant fraction of the primary sequence of a protein and are likely to have a role in protein function. This is the case of the IDRs present in the CREB-binding protein (CBP) [39].

**Figure 4:**
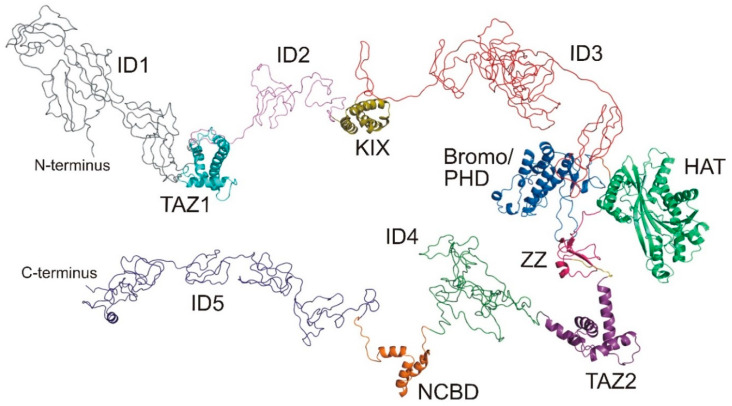
Structural organization of human CBP: ID1 (gray), TAZ1 (cyan, PDB ID: 1U2N), ID2 (violet), KIX (gold, PDB ID: 2LQI), ID3 (red), Bromo and PHD (light blue, PDB ID: 4N4F), HAT (light green, PDB ID: 3BIY), ZZ (pink, PDB ID: 1TOT), TAZ2 (purple, PDB ID: 2KJE), ID4 (green), NCBD (orange, PDB ID: 2KKJ) and ID5 (blue). Disordered regions have been generated using the IntFOLD web resource [40].

Human CBP is a transcriptional regulator found in almost all known cellular pathways and implicated in complex physiological and pathological processes. Its function is mainly based on the interaction with a large variety of transcription factors and other regulatory proteins targeting its intrinsic histone acetyltransferase activity on the chromatin and a broad range of partner proteins. Its domain architecture is shown in Figure 4, along with the structures of the folded domains, determined in recent years by nuclear magnetic resonance spectroscopy (NMR) and X-ray crystallographic methods. In CBP, there are seven domains able to fold independently; four of them require zinc binding to stabilize their tertiary structures: the transcriptional-adaptor zinc-finger-1 (TAZ1) domain, the plant homeodomain (PHD), a zinc-binding domain near the dystrophin WW domain (ZZ), and the transcriptional-adaptor zinc-finger-2 (TAZ2) domain. The other folded domains are the CREB binding domain (KIX), the bromodomain, and the histone acetyltransferase domain (HAT). The nuclear-receptor coactivator-binding domain (NCBD) is intrinsically disordered but folds on interacting with its partner.

Of the 2442 residues that comprise the sequence of CBP, about 60% are in regions of the protein that are outside the structured domains and are likely to be intrinsically disordered. The main role of these IDRs is generally assumed to confer enough flexibility for the assembly of the transcriptional machinery [41] and they are disregarded in high-resolution studies. However, they may be far from just structural linkers and might also provide binding sites for transcriptional regulatory proteins, recruit protein factors and exert interactions. Indeed, the three disordered regions characterized so far by NMR, integrated with other biophysical technologies such as small angle X-ray scattering and mass spectrometry [42,43,44,45], demonstrate that the IDRs provide additional opportunities for CBP to orchestrate its function.

**Figure 5:**
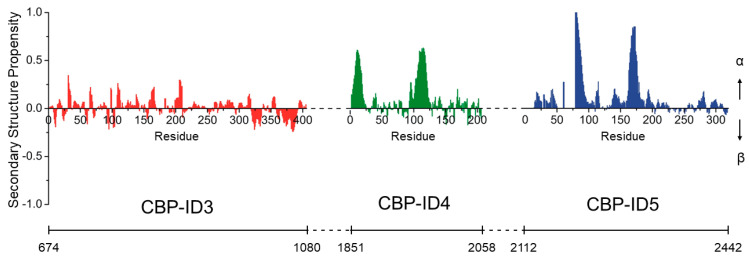
Neighbor-corrected secondary structural propensity (SSP) plots of CBP-ID3 (red), CBP-ID4 (green), and CBP-ID5 (blue) linkers. In the figure, the numbering of each linker has been considered out of the context of the full-length protein while the bottom line reports the corresponding CBP residues’ numbering. The SSP scores were calculated from experimentally measured ^15^N^H^, ^13^C′, ^13^C^α^, and ^13^C^β^ chemical shifts by using the neighbor-corrected structural propensity calculator [48]. Positive and negative values correspond to α-helical and β-sheet propensities, respectively.

The longest linker investigated so far, CBP-ID3 (406 AA), is located between the KIX domain and the bromodomain (residues 674–1080 of CBP). Its amino acid composition is biased toward disorder-promoting amino acids, typically found in intrinsically disordered proteins (IDPs) [46], containing 74 proline (18%), 49 glutamine (12%), and 47 serine (12%) residues. NMR spectroscopy confirms that overall it is disordered but a careful analysis of the dynamics and structural features of the polypeptide, reveals that several regions exhibit small but significant propensities to be structured, which means that it is not a fully random-coil polypeptide. This is also evident from the secondary structural propensity plot (Figure 5) obtained by comparing the experimental chemical shift of the sequentially assigned ^15^N^H^, ^13^C′, ^13^C^α^, and ^13^C^β^ nuclei and the corresponding random-coil chemical shifts [47]. The CBP-ID3 linker has been shown to interact in a specific manner with several proteins and its transient interaction with a novel substrate for CBP-mediated acetylation, the RNA-binding zinc-finger protein 106 (ZFP106), has been characterized [43].

The following linker, CBP-ID4 (207 AA), is located between the TAZ2 and NCBD domains (residues 1851–2058 of CBP). Again, the primary sequence suggests its structurally disordered, with 45 proline (22%) and 34 glutamine (16%). Indeed, it is highly flexible except for the regions encompassing residues 1852–1875 and 1951–1978 which exhibit a high degree of α-helical propensity (Figure 5). Interestingly, proline residues are uniformly distributed along the linker except for these two more structured regions, indicating that they play an active role in modulating the structural features of this CBP fragment [42]. The helices are also likely to be molecular recognition motifs and one of them has been shown to be a target of another disordered protein, the E1A protein from human adenovirus [45].

CBP-ID5 (330 AA) is the C-terminal disordered region of CBP (residues 2112–2442 of CPB). It contains 79 glutamine residues (23%), 18 of which are in a long polyQ tract conferring an α-helical conformation to the region encompassing residues 2189–2211. The region 2287–2297 also samples an α-helical conformation, while the polypeptide is completely disordered elsewhere, punctuated by 45 proline residues (14%) and 37 glycine residues (11%). Also in this case, the IDR revealed a very complex structural and dynamic behavior and its role in regulating the histone acetyltransferase activity of CBP through specific interactions has been proposed [44].

In summary, the atomic resolution investigations of the structural and dynamic properties of these IDRs provide a striking example of how the concept of protein linkers as mere connecting elements between functional domains is far from the truth. Furthermore, the idea of complex proteins as constituted by either folded or disordered regions is a simplification of a continuum between these two extremes, that need to be characterized at atomic resolution. In this endeavor, NMR spectroscopy has a central role.

## 6. Changes of SERCA Protein after Ligand Binding

Sarco/endoplasmic reticulum Ca^2+^-ATPase (SERCA) is a transmembrane protein which plays an important role in maintaining calcium homeostasis in cells. It is a member of the P-type ATPases family, together with Na^+^/K^+^-ATPase, H^+^-ATPase, and H^+^/K^+^-ATPase. It occurs in several isoforms [49] including: (i) SERCA1 (fast-twitch skeletal muscle cells); (ii) SERCA2a (in cardiac or slow-twitch skeletal muscles and brain); (iii) SERCA2b (in vascular smooth muscles, β-pancreatic cells, and other tissues); (iv) SERCA3a (in vascular endothelium, tracheal epithelium, mast cells, and lymphoid cells).

SERCA activity impairment is often connected with chronic diseases and disorders such as cardiovascular diseases, neurodegenerative and muscular disorders, inflammation, diabetes, and cancer [50,51,52]. Therefore, targeting SERCA represents an efficient way in treating various chronic diseases related to calcium signaling. While inhibition of calcium ions pumping into the reticulum could induce apoptosis and cell death, reinforcement of SERCA activity could prevent consequences related to SERCA disorder, caused by oxidative or glycation stress or low expression. Thus, SERCA inhibitors have potential in cancer treatment, while the activators could improve the function of other diseases mentioned above.

In our contribution, we present the results of molecular docking and full optimization of SERCA bound with rutin derivatives (inhibitors) [53] and compound CDN1163 (activator) [54].

The optimal structures of the ligands were obtained by Spartan software (SPARTAN’08 (Wavefunction Inc., Irvine, CA, USA) using the conformer search method and MMFF94 force field. We used the PDB structures 3w5c for E2 state and 4xou for E1 state of SERCA1a. The structures of protein were treated to correct the bonds and hydrogens by means of the software YASARA, ver. 18.12.27 [55]. Both global docking search Global docking and subsequent optimization of the complexes were performed using the AMBER14 force field.

**Figure 6:**
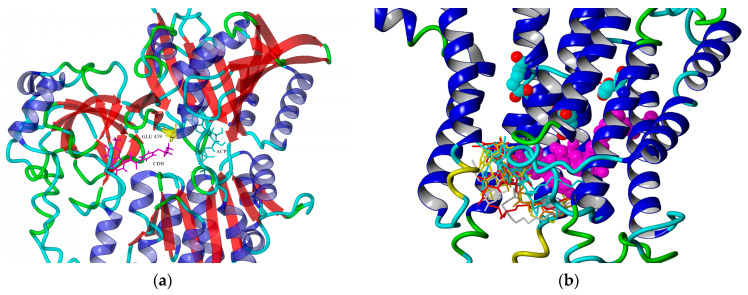
Binding site of CDN1163 in 4xou model of SERCA1a as calculated by YASARA software. CDN1163—magenta, Glu439—yellow and ACP—cyan (**a**); binding modes of rutin derivatives (stick models) in 3w5c model of SERCA1a (**b**). Rutin arachidonate (magenta) shows the position form MD simulation [56]. Glu309, Glu771, and Glu908 are depicted in ball model, element colors.

The positions of rutin derivatives obtained by calculations are shown in Figure 6. right, together with rutin arachidonate as obtained by molecular dynamics simulations [56]. Score values obtained are summarized in Table 2. The most preferred position of CDN1163 is shown in Figure 6 left together with ATP analog and residue Glu439.

During the catalytic process, SERCA undergoes several structural changes connected with nucleotide binding, phosphorylation, cation binding, and protonation. In agreement with our previous results, inhibition effects of rutin derivatives can be related to their ability to affect calcium binding sites in a transmembrane part of SERCA. Compound CDN1163, the beneficial effects of which have been widely proved [54], is known as an allosteric activator of SERCA. However, the exact mechanism and the site of binding are not known so far. We searched for possible way of activation mechanism through the modulatory function of ATP, which was studied by Clausen et al. [57]. Authors found that the mutation of Glu439Ala induced a significant increase in SERCA dephosphorylation rate (E2P→E2 transition) when measured as a function of ATP concentration. A similar effect may be achieved by the interaction of Glu439 with active ligand (here CDN1163, as shown in Figure 6, left). As dephosphorylation is a rate-limiting step (or set of steps) of the second half of the SERCA1a activity cycle [58], this interference could consequently increase the SERCA activity and explain the mechanism of CDN1163 activation of SERCA.

## 7. Should We Have Complexes with Terpyridines?

Chelating metal ions can be a promising approach to designing novel and effective anticancer drugs. In our recent work, several scaffolds were evaluated for their potency as biologically active chelating and ionophoric agents, including quinolones [59,60,61], quinazolines [62,63,64], and thiosemicarbazones [65,66,67,68]. More recently, we have investigated terpyridines (Tpy) that possess strong chelating activity and potential for development [69]. There are many papers reporting complexes with bipyridines and growing in popularity recently, terpyridines. However, there is much less information about the activity of free ligands. From a medicinal chemistry point of view, we have found it interesting to investigate the current level of knowledge of the anticancer activity of terpyridines. This helped us point out some popular but unconfirmed biases and highlight the differences between the mechanisms of activity of ligands and their complexes. Finally, we could design new highly active Tpy derivatives with high selectivity towards cancerous cells.

It is trivial to say that polypyridine systems merit interest for their ability to form complexes with various metal cations. The large fused aromatic scaffold makes the terpyridines and their derivatives typical non-innocent ligands [70]. The contribution of the system to the electronic energy of the central metal leads to a more non-defined oxidative state on this atom, which in turn permits the redox activity of the whole complex. This feature opens the possibility of creating a variety of fascinating applications in areas such as supramolecular chemistry [71], photovoltaic cells [72], pollutant degradation, or catalysis, among others. The low redox barrier that can be observed in Tpy, also supports their biological activity as antitubercular, antiprotozoal inhibitors, and anticancer agents, which is the most abundant bioactivity reported in the literature [69]. Complexes with transition metals have gained attention after the success of cisplatin that was introduced as an anticancer drug and remains in use today after almost 50 years. Similarly, the terpyridines have been investigated as complexes with transition metals, among which first row and selected metals from the next rows of the Periodic table, such as Pt, Pd, and Au, are predominant. Recently, a Ru-complex with a polypyridyl system (TLD1433) has entered clinical trials as a photosensitizer that is used in the photodynamic therapy of bladder cancer [73]. Although no other Tpy-based drugs have been accepted for anticancer therapy, these intense explorations have helped to reveal some important properties of Tpy compounds, such as the mechanism of action and the kinetically controlled dynamics of ligand exchange. One of the prerequisites determining the success of metal-based drugs is a specific three-dimensional configuration and electronic potential, which are unavailable in more typical, purely organic molecules. A wide range of properties, including biological activities, can be obtained in combination with various organic ligands. Increasing attention from the scientific community has resulted in awkward simplification of the investigated space and approaches that have been reported in a wide selection of published reports. Namely, the advantageous activity of the Tpy complexes was typically accepted as a fact. In the majority of reports, free ligands were not involved in the experimental schedule nor the discussions or elucidations of the results. This has resulted in a strongly biased opinion about the biological potency of the Tpy, without a proper scientific basis. Indeed, today it is easier to find a statement that specific Tpy complexes are more active than their ligands than a sound comparison of the data for either. Most strikingly, authors of those more comprehensive reports that have included ligands in the experimental procedures and found them more active than complexes were surprised by their observations.

**Figure 7:**
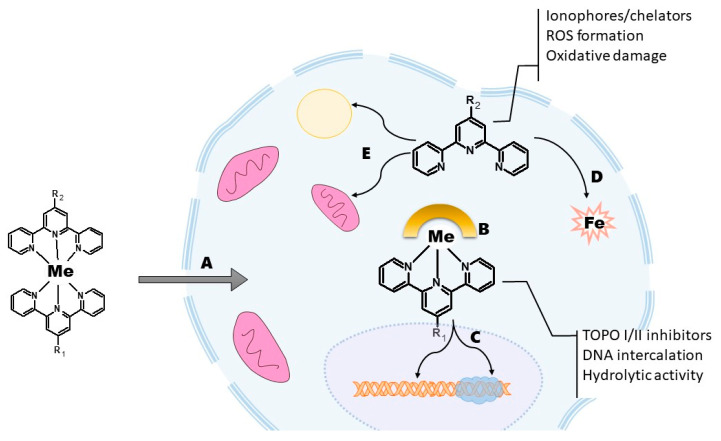
General depiction of intricate interactions between Tpy complexes and cellular environment that may lead to dissociation (**A**) and ligand exchange (**B**). Liberated molecules of Tpy and mono-complexes may further interact with targets exerting their activity in different manner. Complexes tend to intercalate and hamper the topoisomerases (**C**), while free ligands may act as chelating agents and ionophores generating ROS (**D**) and disturb the metabolic balance (**E**).

At the same time, some very active Tpy derivatives have been described. Interestingly their mechanism of action differs substantially from that described for complexes. The more in-depth analysis helped us reveal some facts neglected by many of the reports. Namely, Tpy complexes with transition metals are not as stable as suggested by isolated experiments in laboratory conditions [74,75]. The stability of a complex may alter considerably in a cellular environment burdened by competing ions, pools of different pH, the presence of other ligands, and hydrolytic enzymes. Therefore, the observable effects may result from complex interactions between the molecule of interest with the biochemical matrix and the products of the reactions that take place during those interactions. For example, work published by Grau et al. revealed the importance of dissociation in the biological environment in the antiproliferative activity of the Tpy–Cu complexes [76]. Authors described a 100× increase in activity during prolonged (72 h vs. 24 h) incubation with various cancer cell lines. These observations are in agreement with the strong activity of some Tpy derivatives that were mentioned above. Therefore, interactions between Tpy complexes and cells appear to be complicated, involving different dissociation processes, ligand exchange, chelating and ionophoric activity of the dissociation products as well as generation of reactive oxygen species in Fenton-like reactions (Figure 7) [77].

Another interesting aspect revealed by our research is the difference in the mechanism of activity between Tpy and their complexes [69]. Free ligands and labile first-row metal complexes express ionophoric activity, and strong redox activity, including oxidative damage in DNA, but no activity against topoisomerases. By contrast, the heavy metal complexes have often been described as strong inhibitors of TOPO I/II and DNA intercalators. The same applies to the differences in cell death modes triggered by Tpy and their complexes. Cell cycle blockade during the G1 or S phases is more typical for the ligands while a higher concentration of uncoiled DNA during the phases S-M makes the cell more vulnerable to complexes with transition metals from the second or third row. These findings helped us design novel highly active Tpy derivatives oriented towards their potential cellular targets [78,79,80]. The most effective compounds reached nanomolar levels of activity with good selectivity.

To sum up, serious precautions should be taken in designing an investigation of polypyridine systems such as Tpy and their complexes. The ligands should be considered in the experimental plan for every novel compound as well as other positive and negative controls. There is also an urgent need for a simple but reliable method for assessing the stability of the complexes in the cell. Consequently, the fate of the complexes within the cells or tissues may suggest possible pathways and mechanisms of their activity, that are misleading. Tpy derivatives alone deserve to be considered as promising scaffolds for the design of new anticancer drugs not just as promising substrates for metal-based drugs. Functionalization with fragments introducing novel properties such as lipophilic, basic, and privileged structures opens a route to tailoring Tpy to new biological targets.

## 8. Transition Metal Complexes for Cancer Therapy

Transition metal complexes have been used for cancer therapy for more than 40 years since Rosenberg’s discovery of the antineoplastic properties of cisplatin in 1965 [81], and its FDA approval and introduction to the market in 1978 [82]. Still, nowadays, platinum-based anticancer metallodrugs represent a best-known example of biologically active transition metal complexes. Their unprecedented success triggered the ongoing intensive research in the area of metal-based medicinal chemistry, which resulted in the development of numerous compounds of other d-block metals, which have been studied for various kinds of biological activity (e.g., anticancer) [83]. Some complexes, for example, ruthenium (IT-139) and palladium (TLD1433) agents, have entered clinical trials as new anticancer drugs for the treatment of various types of cancer [84,85].

The distinct advantage that transition metal complexes offer to bioinorganic chemists, lies in the unique possibility of choosing a metal (number of metals, oxidation states) in a specific combination with ligands (coordination modes, substituents). This design strategy enables fine-tuning of various properties, e.g., lipophilicity or biofunctionalization, known to relate to the resulting biological activity [83,86]. Biofunctionalization is based on the introduction of bioactive ligands or carrier-ligand substituents to the structure of newly developed compounds. This underpins the concept of rationally designed multi-targeted metallodrugs, which is, in general, based on a combination of at least two distinct bioactive moieties (species) into a single chemical entity [87]. It has to be noted that there is a difference between the concepts of multi-targeted (metallo)drugs and polypharmacology, the latter of which is based on the detection of at least two different effects (or cellular targets) induced by only one compound.

**Figure 8:**
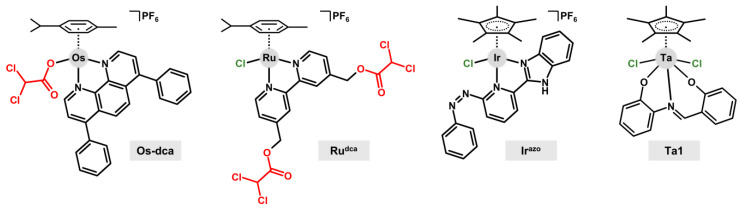
Structural formulas of complexes [Os(η^6^-pcym)(bphen)(dca)]PF_6_ (**Os-dca**), [Ru(η^6^-pcym)(bpy^dca^)Cl]PF_6_ (**Ru^dca^**), [Ir(η^5^-Cp*)Cl(L2^azo^)]PF_6_ (**Ir^azo^**), and [Ta(η^5^-Cp*)Cl_2_(L3)] (**Ta1**); bioactive ligand/substituent dichloroacetate is given in red.

Half-sandwich osmium(II) complex [Os(η^6^-pcym)(bphen)(dca)]PF_6_ (**Os-dca**; Figure 8) represents an example of a multi-targeted transition metal complex [88], combining the cytotoxic Os-based species and dichloroacetate, which is a drug for the treatment of lactic acidosis [77]; pcym = 1-methyl-4-(propan-2-yl)benzene (*p*-cymene), bphen = bathophenanthroline. **Os-dca** was prepared from the co-studied complex [Os(η^6^-pcym)(bphen)Cl]PF_6_ (**Os-Cl**), and these complexes were studied with Ru analogs (**Ru-dca**, **Ru-Cl**) [88].

Solution behavior studies proved that both **Os**-**dca** and **Ru-dca** hydrolyze in the presence of water, which is connected with a release of the [M(η^6^-pcym)(bphen)(H_2_O)]^2+^ metal-based species and bioactive dca ligand. Importantly for the biological studies, **Os**-**dca** is more stable under the experimental conditions used, which is connected with the effective delivery of the bioactive dca ligand to the treated cells. This is not the case for the hydrolytically unstable Ru analog (**Ru**-**dca**). **Os**-**dca** has been shown to be more cytotoxic in vitro than the reference drug, cisplatin, against various human cancer cell lines [88,89]. Of particular note is the nanomolar potency of **Os-dca** (IC_50_ = 0.5 μM) against aggressive MDA-MB-231 triple-negative breast cancer (TNBC) cells compared to the micromolar activity of cisplatin (IC_50_ = 56.0 μM). Also of importance, such high anticancer activity was not connected with toxicity towards various non-cancerous cells (e.g., human embryonic kidney (HEK) 293), pointing out the high and pharmacologically prospective selectivity of **Os-dca**. Multi-targeted complex **Os-dca** also showed anti-metastatic activity in MDA-MB-231 cells (reduced migration, invasion, and re-adhesion) and reversed the Warburg effect, which can be assigned to the released bioactive dca ligand. Besides apoptosis, which is known to be a prominent mode of cell death for the majority of newly developed metallodrugs [82,83,86], oncosis was detected in the cells treated by **Os-dca** [89].

Since **Os-dca** has proved to be a suitable candidate for the development of new metal-based drugs for a hard-to-treat type of breast cancer [88,89], it has also been tested for its anticancer potency towards human breast cancer stem cells (CSC) [90]. Indeed, **Os-dca** exhibited a selective submicromolar effect against CSC, studied in heterogeneous populations of MCF-7 and SKBR-3 human breast cancer cell lines (both 2D cultures and 3D mammospheres were studied), where **Os-dca** even exceeded the reference drug salinomycin. For the studied breast CSC, necroptosis was detected as the mechanism of cell death.

A different strategy of the introduction of dca to the structure of half-sandwich complexes was studied for compounds [Ru(η^6^-pcym)(bpy^dca^)Cl]PF_6_ (**Ru^dca^**; Figure 8) and [Ir(η^5^-Cp*)(bpy^dca^)Cl]PF_6_ (**Ir^dca^**); bpy^dca^ = 2,2′-bipyridine-4,4′-diyldimethanediyl- bis(dichloroacetate), HCp* = pentamethylcyclopentadiene [91]. These model dca-functionalized complexes released the terminal bioactive substituents (i.e., dca) very quickly in the presence of PBS, while co-studied acetate-substituted analogs [Ru(η^6^-pcym)(bpy^ac^)Cl]PF_6_ (**Ru^ac^**) and [Ir(η^5^-Cp*)(bpy^ac^)Cl]PF_6_ (**Ir^ac^**) were adequately stable under these experimental conditions. This allowed us to perform additional experiments in the presence of porcine liver esterase (PLE) for **Ru^ac^** and **Ir^ac^**. The results proved that the ester bonds of these complexes were stable even in the presence of PLE, where the free bpy^ac^ ligand released its acetate substituent. Complexes **Ru^dca^**, **Ir^dca^**, **Ru^ac^**, and **Ir^ac^** were inactive (IC_50_ > 100 μM) against the used human cancer cell lines (e.g., MCF-7 breast adenocarcinoma).

A similar strategy was applied for osmium(II) complex [Os(η^6^-pcym)Cl(L^ind^)]PF_6_ (**Os^ind^**) containing the 2-(1,3,4-thiadiazol-2-yl)pyridine-based ligand bearing the cyclooxygenase inhibitor indomethacin (ind) [92]. This time, the bioactive substituent (i.e., ind) was bound to the carrier chelating ligand through the amide bond, which was cleaved exclusively in the presence of enzyme carboxypeptidase A (from bovine pancreases).

In the case of another series of complexes [Ru(η^6^-pcym)Cl(L1^azo^)]PF_6_, [Ir(η^5^-Cp*)Cl(L1^azo^)]PF_6_ and [Ir(η^5^-Cp*)Cl(L2^azo^)]PF_6_ (**Ir^azo^**), isomeric ligands 2-{5-[(*E*)-phenyldiazenyl]pyridin-2-yl}-1*H*-benzimidazole (L1^azo^) and 2-{6-[(*E*)-phenyl- diazenyl]pyridin-2-yl}-1*H*-benzimidazole (L2^azo^) were used [93]. The complexes were designed to have an azo bond outside the chelating ring to make it accessible for interactions with various relevant biomolecules we planned to study. In the field of anticancer half-sandwich complexes, it is known that the interaction of similar reaction centers, such as the azo bond, with various biomolecules (e.g., NADH coenzyme) disrupts redox homeostasis of the treated cancer cells and consequently, contributes to cancer cell death [83]. In our study, it was observed that NADH and ascorbate were oxidized to NAD^+^ and dehydroascorbate, respectively, in the presence of **Ir^azo^**, which was connected with the azo bond reduction and formation of the hydrazo form of the complex of interest [93]. On the other hand, the interaction with the reduced glutathione (GSH) led to the formation of the dinuclear Ir species, [Ir_2_(η^5^-Cp*)_2_(μ-SG)_3_]^+^, which was connected to the release of L2^azo^ and its reduction to its hydrazo form. Eventually, GSH was oxidized to GSSG. More importantly, experiments performed on **Ir^azo^** with the mixtures of the mentioned biomolecules showed a recovery of ascorbate from dehydroascorbate in the presence of GSH, which was oxidized again to GSSG. This was the first time in the literature that ascorbate was discussed as an intracellular biomolecule, which could play an important role for newly developed half-sandwich metallodrugs.

Finally, for this contribution, [Ta(η^5^-Cp*)Cl_2_(L3)] (**Ta1**; Figure 8) was discussed as the pioneer anticancer tantalum(V) cyclopentadienyl complex and as a new type of anticancer metallodrugs in the field of bioinorganic chemistry; H_2_L3 = 2-{(*E*)-[(2-hydroxy- phenyl)imino]methyl}phenol [94]. The complex exceeded the anticancer potency of cisplatin in the cancer cell lines used (e.g., IC_50_ = 8.6 vs. 20.1 μM in A2780 human ovarian carcinoma cells) and showed the ability to overcome the resistance of cancer cells towards cisplatin, while it was negligibly toxic against non-cancerous cells (MRC-5 fibroblasts, primary culture of human hepatocytes). Relevant processes connected with the mechanism of action were also studied, indicating that **Ta1** induced apoptosis in the treated cancer cells, which is connected with a disruption of mitochondria and induction of the formation of reactive oxygen species.

## 9. Effect of Donor/Acceptor (D/A) Terminal Substituents on Photophysical and Biological Properties of Phenothiazine Derivatives

**Figure 9:**
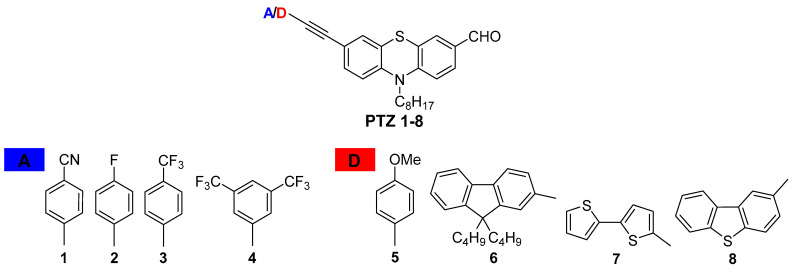
Structures of phenothiazine derivatives **PTZ 1**–**8**.

Phenothiazine (PTZ) and its derivatives are interesting heterocycles that include electron-rich sulfur and a nitrogen atom. Phenothiazine, as a strong electron-donating molecule, is primarily used as a potential building block in the construction of donor-acceptor (D-A) systems. Its non-planar geometry provides exceptionally excellent photophysical properties. Phenothiazine and mostly its substituted derivatives are widely applied in optoelectronics as active components of organic light-emitting diodes (OLEDs) and as photosensitizers in dye-sensitized solar cells (DSSCs), and semiconductors [95,96,97,98,99,100]. They are widely used in the construction of compounds with biological applications, biosensors, or bioimaging agents [101,102,103,104]. The modification of the structure of phenothiazine allows for the design and fine-tuning of their photophysical properties and potential applications in materials science and bioimaging. This work reviews the investigation focused on the synthesis and mainly the influence of donor/acceptor (D/A) terminal units on the photophysical properties of PTZ derivatives **PTZ 1**–**8** (Figure 9).

The phenothiazine derivatives of D/A-π linker-D(PTZ)-A architecture were obtained in a multi-step reaction, including alkylation, formylation, and bromination. The final Sonogashira cross-coupling reaction gave the desired products **PTZ 1**–**8** with satisfactory yields of 40–90% [104,105,106,107]. The phenothiazine aldehydes **PTZ 1**–**8** were designed and synthesized to determine the compounds’ structural relationship and photophysical properties and evaluate their applicability as cellular dyes. The effect of the electron-donating (D) and electron-withdrawing (A) terminal substituents on the synthesized compounds’ absorption and emission properties were observed.

The electron-withdrawing (-CF_3_, -F, -CN) terminal substituents compared to the electron-donating (-OMe, bithienyl, dibenzothienyl, fluorenyl) groups of compounds **PTZ 1**–**8** cause a light redshift of the absorption spectrum of about 10 nm. All compounds **PTZ 1**–**8** are fluorescent, with emissions from 529 to 542 nm in chloroform solutions. The maxima emission of compounds depended on the electronic nature of the terminal units; similarly to absorption spectra, compounds **PTZ 5**–**8** with donor substituents were bathochromically shifted compared to compounds **PTZ 1**–**4**. The molecules possessing fluorine atoms (**PTZ 2**–**4**) exhibit the emission maximum red-shifted with the decreasing number of fluorine atoms indicating the presence of two and one acceptor groups (-CF_3_) lessens the shift to longer wavelengths. This phenomenon caused the reduction of the optical energy band gap between the highest occupied molecular orbital (HOMO) and the lowest unoccupied molecular orbital (LUMO) energy levels. Interestingly, the compound with the cyano group (**PTZ 1**) possessed the same emission maximum and quantum yield of 59% as the compound with one -CF_3_ group (**PTZ 3**), suggesting that the electronic nature of substituents is comparable. The fluorescence quantum yields of compounds PTZ1–8 in chloroform solutions were 38 to 63%. The occurrence of additional fluorines in the phenyl unit, from **PTZ 2** to **PTZ 4,** raises the fluorescence to 63% in the direction **PTZ 2** (40%) → **PTZ 3** (59%) → **PTZ 4** (63%). A parallel rise of quantum yield was observed for the pyrazolo [3,4-*b*]quinoline derivatives bearing different natures of substituents, where the compound with the methyl group possessed a quantum yield of 45%, with trifluoromethyl of 67%, and the highest quantum yield of 90% was noticed for a molecule with trifluoromethyl and additional fluorine atoms [108]. The modest quantum yield of 29% showed the *N*-hexyl-3-phenylethynyl-10*H*-phenothiazine in chloroform solution, and the attachment of the formyl group to the phenothiazine core significantly increased the quantum yield to 39%, suggesting the impact of an extra enhanced donor–acceptor fluorophore [109]. Among the compounds with donor substituents, the strength of donating character reflects in their quantum efficiency, where the compound with the *p*-methoxyphenyl unit (**PTZ 5**) possesses the highest (58%) and with the bithienyl unit (**PTZ 7**) the lowest quantum yield of 38%, and compounds **PTZ 6** and **PTZ 8** comparable quantum efficiency of 49 and 51%, respectively. In the case of compound **PTZ 7**, its low quantum yield can result from a torsion twist between the thiophene rings disturbing the π-electron conjugation.

Additionally, compounds (**PTZ 2**–**5**) with various terminal substituents possessing the highest quantum efficiency were tested as bioimaging probes. It is worth emphasizing that effective fluorescent probes are primarily provoked by intense luminescence. Substituents also have a significant influence on permeability, biological quenching, solubility, aggregation, and bio-affinity to the biological structures. Two compounds of the phenothiazine series **PTZ 2**–**5,** namely, **PTZ 2** and **PTZ 5** with acceptor and donor terminal units, respectively, showed promising potential as fluorescent probes for staining living cells. It was shown that the electronic character of substituents in the compounds that were investigated had a significant impact on the penetration of the biological membranes. The photophysical characteristics of the compounds indicated that the electron ability of terminal units has a considerable influence on their photophysical properties and, thus, their suitability for applications in optoelectronics and bioimaging.

## 10. Preparation and Characterization of Pullulan-Enriched Polymer-Ceramic Composites

In recent years, there has been growing interest in new functional materials for use in regenerative medicine. The market for biomaterials will grow due to the increasing prevalence of bone, cardiovascular, and skin diseases. Biomaterials, which are used for various implants, or in plastic surgery and wound healing, may provide the solution for this growing issue. Biomaterials are designed to coexist with biological systems and thus are useful for treatment, as well as diagnosis and replacement of a complete or partial tissue or organ. In addition, the materials can be derived from nature or artificially manufactured, to replace or support tissue function. Moreover, the functional task of biomaterials is to interact with biological systems in various types of medical instruments and devices [110,111].

One of the most created biomaterials is composites. Biocomposites exhibit a number of advantages, including flexibility, strength, and the possibility of individual, personalized design. In addition, mechanical reliability is magnified compared to monolithic materials. However, one of the biggest disadvantages of composite materials is the difference in properties depending on the used method for their preparation [112,113]. Importantly, nature has many composites, both polymer–ceramic and polymer–polymer. Bone tissue is a biocomposite of the polymer–ceramic type, which consists of an organic matrix, such as collagen or proteins combined with nanometer-sized grains of hydroxyapatite. The material thus formed strengthens the bone. On the other hand, soft tissues are polymer–polymer biocomposites, consisting of collagen fibers immersed in an organic matrix [114].

Ceramic materials are used to create biocomposites as they have high porosity, enabling tissue ingrowth and a permanent bond between the implant and the tissue. In addition, they exhibit high compressive strength, as well as abrasion resistance and high resistance to corrosion in the tissue environment. These materials can be also sterilized with no changes in their properties. However, they are characterized by rather high brittleness [115]. Increasing attention is being focused on calcium phosphate ceramics including hydroxyapatite and brushite. One of the biomaterials that is used in hard tissue repair is brushite (DCPD). It is dicalcium phosphate dihydrate, CaHPO_4_·2H_2_O, which is formed in phosphorite deposits, soil, and human calculi [116,117]. Several in vitro and in vivo studies have suggested that brushite, along with other calcium phosphates, plays a key role as an intermediate phase in the crystallization of more stable hydroxyapatite [118,119]. Brushite is also currently being investigated as a cement for bone substitute materials. Studies showed that brushite cements are well tolerated by the bone environment, as resorption of brushite occurs after the new bone is formed. Furthermore, these cements show good biocompatibility and no inflammatory effect [120]. Additionally, brushite can form composites with collagen or silk. These composites have excellent biological and osteoconductive properties through the protein–brushite combination [121,122].

The matrix of composites can be polymers, especially aliphatic carbonates, polyorthoesters, synthetic polyurethanes, and synthetic amino acids. They are used in the manufacture of implants and medical devices. Moreover, such polymer matrices act as carriers for active substances; for example, drugs, which are delivered to the human body in a controlled manner. The most commonly used polymers are polyethylene glycol (PEG) and polyvinylpyrrolidone (PVP). PEG is synthetic, linear, or branched polyester with one or two hydroxyl groups. It is a condensation polymer of ethylene oxide and water [123]. PEG is the most commonly used non-ionic polymer in drug delivery systems. In recent years, the American Food and Drug Administration (FDA) has recognized this polymer as harmless to the body, and therefore allowed for internal use in research or biomedical applications [124]. Another polymer, polyvinylpyrrolidone (PVP), also known as povidone, is obtained by the polymerization of vinylpyrrolidone and consists of polar amide groups and non-polar ethylene groups. It is soluble in water, alcohols, aromatic hydrocarbons, and halogenated hydrocarbons, as well as in organic acids [125], and has good thermal and chemical resistance and good mechanical properties. PVP is a biocompatible and non-toxic polymer that is used to produce hydrogels [126].

Increasingly, proteins, or carbohydrates, are being added to composite biomaterials to impart new functionalities to the material. One natural, hydrophilic polysaccharide is pullulan. It is produced by the fungus *Aurobasidium pullulans* and is used in hydrogel matrices [127,128]. It exhibits excellent biocompatibility and has been approved by the FDA for use in the pharmaceutical and food industries. Pullulan is also suitable as a component of ceramic-polymer composites, which are promising carrier systems for active substances [129].

To conclude, the growing demand for biomaterials constantly presents new challenges and opportunities for researchers. Combining different materials such as polymers with ceramic materials may result in a composite with new and improved physicochemical, mechanical, and also application properties. The enrichment of composites with polysaccharides will additionally give them biocompatibility with human tissues and find use as potential carriers of active substances.

## Figures and Tables

**Table 1:** Representation of selected applications of Langmuir balance in studies performed for development of drugs found in the available bibliography in the decade 2012–2021.

Application Field	Number of Papers [%]
Drug chemistry	20.97
Drug analysis	20.11
Drug technology	15.63
Drug synthesis	15.62
Pharmaceutical chemistry	11.21
Pharmaceutical formulation	6.81
Drug delivery	3.68
Pharmacology	2.69
Pharmacokinetics	2.62
Biopharmacy	0.65

**Table 2:** Score values of rutin derivatives compared with their inhibition activities to SERCA.

Rutin Derivative	Abbreviation	Corresponding Acyl	IC_50_ [µM] ^1^	Score
rutin palmitate	R16	Palmitoyl	64 ± 12	−8.3
rutin stearate	R18	Stearoyl	35 ± 6.5	−9.7
rutin oleate	R18:1	Oleoyl	50 ± 8.5	−9.8
rutin linoleate	R18:2	Linoleoyl	25 ± 5.5	−9.6
rutin linolenate	R18:3	a-linolenoyl	62 ± 9	−9.5
rutin arachidonate	R20:4	Arachidonoyl	23 ± 6.5	−11.0
rutin erucate	R22:1	Erucoyl	50 ± 8	−9.5

^1^ Values from [53].

## Data Availability

Not applicable.
